# Nudix Hydroxylase 15 Mutations Strongly Predict Thiopurine-Induced Leukopenia Across Different Asian Ethnicities: Implications for Screening in a Diverse Population

**DOI:** 10.3389/fmed.2022.880937

**Published:** 2022-08-05

**Authors:** Xin-Hui Khoo, Shin Yee Wong, Nik Razima Wan Ibrahim, Ruey Terng Ng, Kee Seang Chew, Way Seah Lee, Zhi Qin Wong, Raja Affend Raja Ali, Shahreedhan Shahrani, Alex Hwong-Ruey Leow, Ida Normiha Hilmi

**Affiliations:** ^1^Division of Gastroenterology and Hepatology, Department of Medicine, University of Malaya Medical Centre, Kuala Lumpur, Malaysia; ^2^Clinical Research Centre, Pantai Hospital Kuala Lumpur, Kuala Lumpur, Malaysia; ^3^Department of Hepatology and Gastroenterology, Hospital Selayang, Selangor, Malaysia; ^4^Department of Paediatrics, Faculty of Medicine, University Malaya, Kuala Lumpur, Malaysia; ^5^Gastroenterology Unit, Department of Medicine, Faculty of Medicine, Universiti Kebangsaan Malaysia, Kuala Lumpur, Malaysia; ^6^Department of Medicine, Faculty of Medicine, University of Malaya, Kuala Lumpur, Malaysia

**Keywords:** *NUDT15*, inflammatory bowel disease, leukopenia, genetic polymorphism, thiopurines

## Abstract

**Background and Aims:**

Thiopurines, which are immunosuppressive drugs for maintaining remission for inflammatory bowel disease, are known to cause myelotoxicity in patients with Nudix Hydroxylase 15 (*NUDT15*) genetic variants in some Asian countries with monoethnic populations. We aimed to investigate the association of *NUDT15* variants with leukopenia in a multiethnic population in Southeast Asia.

**Methods:**

Patients with a confirmed diagnosis of inflammatory bowel disease were recruited. We collected demographic and clinical characteristics and whole blood counts before and after initiating thiopurines. Thiopurine S-methyltransferase (*TPMT*) and *NUDT15* genotypes were analyzed with the single nucleotide polymorphisms (SNPs) genotyping assay. Leukopenia was defined as a white blood cell (WBC) count < 3,000/μl.

**Results:**

In this study, 19 (18.6%) of the 102 patients who had adequate thiopurine therapy experienced leukopenia, 11 patients (57.9%) had *NUDT15* c.415C > T variants, 2 patients (10.5%) had *NUDT15* c.52G > A variants while one (5.3%) had a *TPMT* variation. Individually, *NUDT15* c.415C > T had a sensitivity and specificity of 57.9% and 94.0% (odds ratio [*OR*] = 21.45, 95% *CI* 5.94–77.41, *p* < 0.001), respectively, for predicting thiopurine-induced leukopenia, while *NUDT15* c.52G > A was only observed in patients with leukopenia. As compared with patients with wild-type *NUDT15*, both *NUDT15* variations had a combined sensitivity and specificity of 68.4% and 94%, respectively (*OR* = 33.80, 95% *CI* 8.99–127.05, *p* < 0.001), for predicting thiopurine-induced leukopenia as well as a shorter onset to leukopenia (median onset [months] 2.0 vs. 5.5; *p* = 0.045). Sub-group analysis showed that both *NUDT15* variations were strongly associated with leukopenia among the Chinese and Indians but not among the Malays.

**Conclusion:**

*Nudix Hydroxylase 15* variants strongly predicted thiopurine-induced leukopenia across a multiethnic Southeast Asian population, particularly among the Chinese and Indians.

## Introduction

Inflammatory bowel disease (IBD) is emerging rapidly in Asia, including Malaysia (1). The mean incidence of IBD in Malaysia increased from 0.07 to 0.69 per 100,000 person-years over the past two decades (2). Despite a paradigm shift in the management of IBD toward the early use of biologics in complicated cases or those with a severe phenotype, the Malaysian National IBD database from 2015 showed that biologics were only used in 2.5% of patients with IBD, whereas another 39.8% of patients were on thiopurines (unpublished data). This pattern of IBD therapy is likely to be similarly seen in many countries with limited resources where immunomodulators, such as thiopurines, remain an important therapy of choice for the maintenance of remission in patients with the steroid-dependent disease.

After oral administration, thiopurines undergo a complex series of metabolic pathways. Azathioprine (AZA), the most commonly used thiopurine, is converted to 6-mercaptopurine (6-MP), which is then enzymatically converted to its active metabolite, deoxy-6-thioguanosine 5′ triphosphate (6-TGN), through successive enzymatic conversion by hypoxanthine-guanine phosphoribosyl transferase (HGPRT) and inosine monophosphate dehydrogenase (IMPDH) (3, 4). The 6-TGN metabolite is the predominant active compound responsible for thiopurine therapeutic efficacy. During cell division, 6-TGN incorporates into the double-stranded DNA of the cellular nucleus, resulting in disruption of nucleic acid synthesis and cell apoptosis (4).

Tolerance to thiopurines varies widely among different populations based on their pharmacogenetic profile. Intolerance to thiopurines, such as potentially life-threatening myelotoxicity, has been reported in up to 28% of individuals exposed to AZA (5). Variations in the thiopurine S-methyltransferase (*TPMT*) gene are widely associated with an increased incidence of thiopurine-associated myelotoxicity (6). Despite that thiopurine-induced myelotoxicity was more commonly seen among Asians than Whites (7), the prevalence of *TPMT* gene variations is reportedly to be uncommon in Asian populations (8). This has led to the discovery of a novel predisposing gene, the Nudix Hydroxylase 15 (*NUDT15*), which was initially identified in a Korean genome-wide association study (9). Studies from China, Japan, Hong Kong, Singapore, and India have shown a significant association between *NUDT15* variations (particularly c.415C > T) and thiopurine-induced myelotoxicity as well as confirming the lack of utility of *TPMT* variants in predicting thiopurine-induced myelosuppression (10–15).

Unlike the populations of China, Japan, Korea, and Hong Kong, which are largely monoethnic, Malaysia is unique, in that it is made up of three large ethnic groups: Malays, Chinese, and Indians (16). Indians from Malaysia are mainly from South India and Sri Lanka and are generally homogenous. Similarly, the Chinese in Malaysia, mainly from Southern China, are also homogeneous. The Malays, on other hand, are more heterogeneous due to interracial marriages with both the Chinese and Indian ethnic groups, despite being largely Austronesian in origin. Since thiopurines are commonly used as immunosuppressants in Malaysian patients with IBD, we aimed to investigate the association between *NUDT15* variations (c.415C > T and c.52G > A) and leukopenia in this multiethnic population. We also ascertained whether there were any differences in *NUDT15*-associated leukopenia among the three ethnic groups.

## Materials and Methods

### Study Population

Patients with a confirmed diagnosis of IBD were recruited from the University of Malaya Medical Centre (UMMC), Hospital University Kebangsaan Malaysia (HUKM), and Hospital Selayang from March 2017 to February 2021. The diagnosis of IBD was made based on standard clinical, endoscopic, radiological, and histological criteria. Demographic and clinical characteristics of the patients, such as gender, age, age at diagnosis, IBD subtype (Crohn’s disease [CD], ulcerative colitis [UC], or IBD-unclassified [IBD-U]), duration, and dosage of thiopurine therapy, weight, concomitant medications, and blood results, were collected upon recruitment into the study. Blood was collected from the recruited patients during their routine blood check. DNA was extracted for genetic analysis. Informed consent was obtained from all patients prior to data and sample collection. This study was approved by the University of Malaya Medical Centre Ethics Committee (MREC-ID: 2017109-5662).

Patients who were never exposed to thiopurines and those with insufficient exposure to thiopurines (maximum dose < 1.0 mg/kg/day) without signs of myelotoxicity were excluded from the final analysis. Leukopenia was defined as a white blood cell (WBC) count < 3,000/μl.

### Monitoring of Leukopenia

After thiopurine therapy was commenced, full blood counts were reviewed weekly in the first month and then every 3 months after dose escalation. Typically, the starting dose of AZA was 50 mg/day in our centers and was escalated subsequently to 1.5–2 mg/kg/day. Patients who developed nausea on AZA were switched to 6-MP at doses up to 1.5 mg/kg/day.

### DNA Extraction

Genomic DNA was extracted from whole blood by using the DNeasy^®^ Blood & Tissue Kit (Qiagen, Hilden, Germany) according to the manufacturer’s protocol. DNA quality and concentration were validated with the Nanodrop™ 2000 Spectrophotometer (Thermo Fisher, MA, United States) to ensure sufficient quantity and quality of the sample before gene amplification through polymerase chain reaction (PCR).

### Single Nucleotide Polymorphisms Genotyping Analysis of Nudix Hydroxylase 15 and Thiopurine S-Methyltransferase Single Nucleotide Polymorphisms

Genotyping of *NUDT15* c.415C > T (rs116855232), *NUDT15* c.52G > A (rs147390019), *TPMT* c.719A > G (rs1142345), and *TPMT* c.460G > A 9(rs1800460) were performed with the Taqman^®^ SNPs Genotyping Assay (Applied Biosystems, MA, United States) according to the manufacturer’s protocol. Briefly, 10 ng of genomic DNA was aliquoted into a Fast Optical 96-well Microplate and mixed with a PCR mixture containing TaqMan Genotyping Master Mix, TaqMan SNPs Genotyping Assay, and an appropriate amount of ultrapure water. Amplification of the sample was performed using the StepOne^®^ Real-Time PCR machine (Applied Biosystem, MA, United States). The PCR thermal cycling was as followed: initial denaturing at 95°C for 10 s, followed by 95°C for 15 s for 50 cycles, and extension at 60°C. SNPs status was analyzed with Allelic Calling Function.

### Statistical Analysis

A descriptive analysis was carried out on the demographics, clinical, and genetic characteristics. Continuous variables were expressed as median (interquartile range, IQR) and compared using a non-parametric test. Categorical variables were expressed as frequencies and compared using the *X*^2^ test or Fisher’s exact test. The value of *p* < 0.05 was considered significant. All analysis was carried out with SPSS Statistics v.26.0 (IBM, New York, NY, United States).

## Results

### Demographics, Clinical, and Genetic Characteristics

A total of 140 patients with a confirmed diagnosis of IBD were recruited. The demographic and clinical characteristics of the patients are shown in Table 1. The male:female ratio was 6:4, while the ethnicity composition was 40.7% Indian, 32.9% Chinese, and 26.4% Malay. Two-thirds (66.4%) of the patients have CD, 31.4% have UC, while the remaining 2.1% have IBD-U. All the 140 patients had *TPMT*/*NUDT15* mutation analysis performed.

**TABLE 1:** Demographics, clinical, and the thiopurine S-methyltransferase (*TPMT*)/Nudix Hydroxylase 15 (*NUDT15*) genotypes in 140 patients with inflammatory bowel disease (IBD).

Characteristics (*n* = 140)		
Age of Diagnosis, year [median (IQR)]		25.0 (16.0)
**Weight, kg [median (IQR)]**		56.3 (20.0)
Gender, n (%)	Male	81 (57.9)
	Female	59 (42.1)
Ethnicity, n (%)	Malay	37 (26.4)
	Chinese	46 (32.9)
	Indian	57 (40.7)
IBD Type, n (%)	Crohn’s disease	93 (66.4)
	Ulcerative Colitis	44 (31.4)
	IBD-Unclassified	3 (2.1)
**Thiopurine exposure, n (%)**		118 (84.3)
*TPMT* c.719A > G, n (%)	Wildtype (A/A)	135 (96.4)
	Heterozygous variant (A/G)	5 (3.6)
*TPMT* c.460G > A, n (%)	Wildtype (G/G)	140 (100.0)
*NUDT15* c.415C > T, n (%)	Wildtype (C/C)	122 (87.1)
	Heterozygous variant (C/T)	17 (12.1)
	Homozygous variant (T/T)	1 (0.7)
*NUDT15* c.52G > A, n (%)	Wildtype (G/G)	138 (98.6)
	Heterozygous variant (G/A)	2 (1.4)

*IBD, inflammatory bowel disease.*

The overall frequency of *TPMT* c.719A > G (rs1142345) variation was 3.6% (*n* = 5), while *TPMT* c.460G > A was non-polymorphic in our study cohort. The overall frequency of *NUDT15* c.415C > T and *NUDT15* c.52G > A variations were 12.8% (*n* = 18) and 1.4% (*n* = 2), respectively.

### Association Between Thiopurine S-Methyltransferase and Nudix Hydroxylase 15 Variants and Leukopenia

In this study, 119 of the 140 patients were treated with thiopurines. Of these, 17 patients had inadequate exposure (maximum dose < 1.0 mg/kg/day) during the time of recruitment. Thus, only 102 patients with sufficient exposure to thiopurine were included for the final analysis of the association between genetic variations and leukopenia.

Of the 102 patients, 19 (18.6%) patients experienced leukopenia during treatment with thiopurines. There were no significant differences in IBD subtype, age, body weight, gender, ethnicity, and thiopurine dosage (Table 2). Thiopurines were discontinued at a median of 5.0 months (IQR 8.0) in patients who developed leukopenia. Four of the five patients who were found to have *TPMT* c.719A > G (rs1142345) variation were analyzed for leukopenia. Only one patient (25%) developed leukopenia. The remaining 18 patients who developed leukopenia had wild-type *TPMT* variations.

**TABLE 2:** Predictive factors of patients with or without thiopurine-induced leukopenia.

	No leukopenia (*n* = 83)	leukopenia (*n* = 19)	Odds ratio (confidence interval)	*p*-value
Age of diagnosis, y [Median (IQR)]	24.0(9−63)	26.0(10−55)	−	0.663
Weight, kg [Median (IQR)]	55.4(30−118)	55.0(25−94)	−	0.065
**Gender, n (%)**				
Male	54 (65.1)	8 (42.1)	−	–
Female	29 (34.9)	11 (57.9)	2.013(0.53−7.65)	0.305
Ethnicity, n (%)				.
Malay	21 (25.3)	4 (21.1)	−	–
Chinese	27 (32.5)	7 (36.8)	0.619(0.10−3.74)	0.601
Indian	35 (42.2)	8 (42.1)	1.169(0.32−54.92)	0.846
Thiopurine dosage, mg/kg/day [median (IQR)]	1.58 (0.59)	1.53 (0.54)	−	0.445
Thiopurine duration, months [median, (IQR)]	36.0 (60.0)	5.0 (8.0)		<0.001
***TPMT* c.719A > G, n (%)**				
Wildtype (A/A)	80 (96.4)	18 (94.7)		
Heterozygous variant (A/G)	3 (3.6)	1 (5.3)		
***TPMT* c.460G > A, n (%)**				
Wildtype (G/G)	83 (100.0)	19 (100.0)		–
***TPMT* variation**				
Yes	3 (3.6)	1 (5.3)	4.19(0.32−54.92)	0.275
No	80 (96.4)	18 (94.7)		
***NUDT15* c.415C > T, n (%)**				
Wildtype (C/C)	78 (94.0)	8 (42.1)		
Heterozygous variant (C/T)	5 (6.0)	10 (52.6)		
Homozygous variant (T/T)	0 (0.0)	1 (5.3)		
***NUDT15* c.52G > A, n (%)**				
Wildtype (G/G)	83 (100.0)	17 (89.5)		
Heterozygous variant (G/A)	0 (0.0)	2 (10.5)		
***NUDT15* variation**				
Yes	5 (6.0)	13 (68.4)	41.49(9.55−180.28)	<0.001
No	78 (94.0)	6 (31.6)		

In contrast, *NUDT15* variations were significantly more common in individuals who developed leukopenia (odds ratio [*OR*] = 41.49, 95% *CI*, 9.55–180.28, *p* < 0.001) after thiopurine therapy. Ten of the 19 patients with leukopenia were heterozygous for *NUDT15* c.415C > T while one patient was homozygous for *NUDT15* c.415C > T variation. The patient who was homozygous for *NUDT15* c.415C > T was a 25-year-old Chinese male (78 kg) who was started on 100 mg of AZA (1.3 mg/kg). The patient developed profound neutropenic sepsis (WBC count 6 × 10^9^/L and neutrophil count unrecordable) and alopecia requiring antibiotics and granulocyte colony-stimulating factor (GCSF) for 2 weeks. Other two patients had *NUDT15* c.52G > A (Table 2).

No co-occurrences of *TPMT* and *NUDT15* variations were observed in our cohorts. No *TPMT* or *NUDT15* variants were identified in five (26.3%) of the 19 patients who developed thiopurine-induced leukopenia.

Individually, *NUDT15* c.415C > T had a sensitivity and specificity of 57.9 and 94.0%, respectively, (*OR* = 21.45, 95% *CI* 5.94–77.41, *p* < 0.001) in predicting thiopurine-induced leukopenia while *NUDT15* c.52G > A were only observed in patients with leukopenia. In combination, both *NUDT15* variations had a sensitivity and specificity of 68.4 and 94%, respectively, of predicting thiopurine-induced leukopenia (*OR* = 33.80, 95% *CI* 8.99–127.05, *p* < 0.001) (Table 3).

**TABLE 3:** Sensitivity and specificity of combined *NUDT15* SNPs c415.C > T and c.52G > A in predicting thiopurine-induced leukopenia.

*NUDT15* SNPs	Sensitivity	Specificity	OR (95%CI)	*p*-value
c.415C > T	57.9%	94.0%	21.45 (5.94 - 77.41)	<0.001
c.52G > A	100.0%	83.0%	–	–
Combined	68.4%	94.0%	33.80 (8.99- 127.05)	<0.001

**FIGURE 1:**
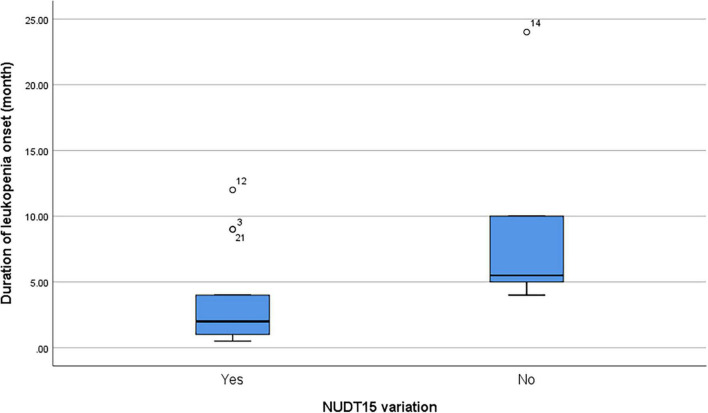
Duration of thiopurine treatment in patients before the onset of leukopenia.

Among patients with leukopenia (*n* = 19), the presence of *NUDT15* variations (*n* = 13 including both *NUDT15* c.415C > T and *NUDT15* c.52G > A) also predicted a shorter onset to leukopenia (median = 2 months and range = 0.5–12 months) compared to patients with wild-type *NUDT15* (median = 5.5 months, range = 4–24 months; *p* = 0.045) (Figure 1).

### Ethnicity and Nudix Hydroxylase 15 Variations

The sub-group analysis was conducted for the three different ethnic groups in Malaysia separately. The *NUDT15* variations (combination of c.415C > T and c.52G > A) were shown to be strongly associated with leukopenia among Chinese and Indians. In Malay patients, however, only 1 patient (25%) with leukopenia had *NUDT15* mutations. This was not statistically significant due to the small number of patients (Table 4).

**TABLE 4:** The *NUDT15* variations in patients with IBD with and without leukopenia among the three major ethnic groups in Malaysia.

Ethnics	Non-leukopenia	Leukopenia	*p*-value
**Malay, n (%)**			
*NUDT15* wildtype	20 (95.3)	3 (75.0)	0.300
*NUDT15* variants	1 (4.7)	1(25.0)	
c.415C > T	1 (4.7)	1(25.0)	
c.52G > A	0 (0.0)	0 (0.0)	
**Chinese, n (%)**			
*NUDT15* wildtype	25 (92.6)	1 (14.3)	<0.001
*NUDT15* variants	2 (7.4)	6 (85.7)	
c.415C > T	2 (7.4)	4(57.1)	
c.52G > A	0 (0.0)	2 (28.6)	
**Indians, n (%)**			
*NUDT15* wildtype	33 (94.3)	2 (25.0)	<0.001
*NUDT15* variants	2 (5.7)	6 (75.0)	
c.415C > T	2 (5.7)	6 (75.0)	
c.52G > A	0 (0.0)	0 (0.0)	

## Discussion

Thiopurine is an important therapeutic agent in the armamentarium of autoimmune conditions in countries where reimbursement for expensive therapies, such as biologics, remains limited. Unfortunately, they are associated with a significant risk of complications, and among the most life-threatening is leukopenia, which is closely linked with the individual’s pharmacogenetic profile. *TPMT*, the first identified gene to be strongly associated with thiopurine-induced myelotoxicity, was discovered mostly among White populations. Studies have shown that *TPMT* genotyping prior to thiopurine therapy reduces the risk of adverse effects without compromising treatment efficacy (17–20). However, the low prevalence of *TPMT* variations in Asians limited the utility of *TPMT* genotyping. Many recent studies from Asia have shown that the frequency of *TPMT* mutation is about 1.2–2.0% in the Asian population and was not predictive of the occurrence of leukopenia in this population (9–11, 21–23). In contrast, most studies from this region have shown that the incidences of allelic mutation in *NUDT15*, mainly c.415C > T, range between 7.4 and 25.7% in Asian populations and strongly predict leukopenia (11–15, 21, 22).

Our study shows that the prevalence of *NUDT15* c.415C > T is 12.8% in Malaysian patients with IBD, similar to a study conducted in Singapore with similar major ethnicities (14). In our multiethnic population, the predictive sensitivity of *NUDT15* c.415C > T for leukopenia was 57.9%, which was within the range of other studies with homogeneous ethnicity including Chinese (49–75%) (10, 11, 24), Koreans (40–90%) (9, 22, 25), and Japanese (44–60%) (12, 13). In addition, *NUDT15 c.415C* > *T* was also reported to be associated with thiopurine-induced myelotoxicity in European and native American population (20, 26, 27), although the prevalence was much lower compared with Asian populations. The predictive sensitivity of *NUDT15 c.415C* > *T* was 3.27% in general Europeans (26) and 4.0% in non-Finnish Europeans (27). Other variants of *NUDT15* were shown to have a higher association with the European population. Walker et al. (27) reported that 9.5% of non-Finnish European patients with IBD with thiopurine-induced myelotoxicity had *NUDT15* variants, with the majority having p.Gly17_Val18del (*NUDT15 c.37_42delGGAGTC*) variant (19/35) (27). Schaeffeler et al. (26) on the other hand reported a higher prevalence of NUDT15 c.*7G > *A* (7.48%) in their general European population (26). In both studies, *TPMT* variants were still mainly responsible for their thiopurine-induced myelotoxicity.

The other variant, *NUDT15* c.52G > A, had a much lower prevalence and was only found among the Chinese ethnic group. Both cases were leukopenic, which resulted in a sensitivity of 100%, but due to the very small numbers, this should be interpreted with caution. Sutiman et al. (14) reported that two heterozygous c.52G > A mutations in the Malays and Chinese populations were shown to have trends of the lower nadir of white cell count and absolute neutrophil count; however, no statistical significance was seen due to the low prevalence and small sample size (14). A large cohort study conducted involving the Han Chinese population reported a prevalence of 4.5% and a sensitivity of 47.9% in predicting leukopenia for this variant (28).

Combining these two, *NUDT15* variations strongly predicted leukopenia in our population, where individuals with the variants were 41 times more likely to develop leukopenia than those without. Patients with *NUDT15* variations were also shown to correlate with a shorter onset of leukopenia compared with patients with wild-type *NUDT15*. Yang et al. (9) reported that *NUDT15* C > T was found to have a higher correlation with the onset of leukopenia at less than 8 weeks. Other studies concluded that *NUDT15* c.415C > T variation was associated with early onset leukopenia (29, 30). Interestingly, *NUDT15* variants were not identified in about one-third of patients who developed leukopenia in the current study. Schaeffeler et al. (26) also showed that about 38% of thiopurine-induced myelotixicty patients did not have *TPMT* or *NUDT15* variations and comedication toxicity was also excluded (26). We postulate that this may be due to other novel mutations within the *NUDT15* gene or other genes that have not yet been discovered.

In view of our multiethnic population, we also ascertained specifically whether the *NUDT15* variants predicted leukopenia in all the major ethnic groups in Malaysia or only in certain ethnic groups. The present study showed clearly that *NUDT15* variants strongly predicted leukopenia not only in the Chinese ethnic group but also in the Indian ethnic group. This is somewhat surprising as the ethnic Indians in Malaysia are thought to be genetically diverse when compared with other homogeneous East Asian populations (Japanese, Koreans, and Chinese). Nevertheless, the finding is completely consistent with studies from India, which showed that the *NUDT15* variants also strongly predicted leukopenia (15). Numerically, *NUDT15* variations did not seem to be highly predictive in Malays (only 1 out of the 4 patients with leukopenia had the variant), and only 2 out of 25 Malay individuals in this study had the variants. Compared with the Chinese and Indians, which had a predictive percentage of 87.5 and 75%, respectively, *NUDT15* variants did not have a significant contribution to the leukopenia in Malays. However, this could also be due to the small sample size and the lower number of patients with leukopenia of Malay ethnicity.

Inflammatory bowel disease is emerging rapidly in Malaysia. The highest prevalence is among the Indian ethnic group, but it is reported in all the major ethnicities (31). As mentioned previously, thiopurines remain an important therapeutic option in our population and will probably continue to be one as they are relatively cheap, efficacious, and familiar with physicians as compared with other immunomodulators, such as methotrexate, tacrolimus, and mycophenolate. Therefore, optimizing its use will remain a relevant issue in the near future in Asia. Recently, the Clinical Pharmacogenetics Implementation Consortium Guidelines also recommended *NUDT15* genotyping in the Asian population if available (32). Unlike *TPMT*, which was widely recognized, currently, there is no regulation or enforced screening of *NUDT15* despite much research having proven the relevance of these genetic factors prior to starting thiopurine therapy. From our study, we encourage all patients regardless of ethnicity to be screened for the identified *NUDT15* mutations in view of the high sensitivity and specificity (14, 15, 30). Life-threatening side effects in our patient who was homozygous for the *NUDT15* variant could have been avoided had screening been carried out prior to commencing therapy.

The main limitation of our study is the relatively small sample size. Future studies involving larger cohorts, particularly among the Malay ethnic group, are needed. Another limitation is that this study focused only on two of each *NUDT15* variant mainly due to the high prevalence reported in previous studies. Other *NUDT15* variations, such as c.457C > T, c.37_42delGGAGTC, and c.55_56insGAGTCG, were not included in this study, owing to the low frequency of these variants reported previously in Singapore (14, 20). Therefore, potential contribution of these variants may be missed out in our cohort.

## Conclusion

In conclusion, *NUDT15* variants strongly predicted thiopurine-induced leukopenia across a multiethnic Asian population, particularly among the Chinese and Indians. Screening for these variants should be mandatory prior to commencing thiopurine therapy.

## Data Availability Statement

The original contributions presented in the study are included in the article/Supplementary Material, further inquiries can be directed to the corresponding author/s.

## Ethics Statement

The studies involving human participants were reviewed and approved by the Medical Research Ethics Committee, University of Malaya Medical Centre. The patients/participants provided their written informed consent to participate in this study.

## Author Contributions

IH, WL, and RR: study conception. NI, SS, RN, KC, ZW, and AL: acquisition of data. X-HK and SW: analysis and interpretation of data and drafting the manuscript. IH, WL, RR, NI, SS, RN, KC, ZW, and AL: revision for important intellectual content. All authors approved the final version of the manuscript.

## Conflict of Interest

The authors declare that the research was conducted in the absence of any commercial or financial relationships that could be construed as a potential conflict of interest.

## Publisher’s Note

All claims expressed in this article are solely those of the authors and do not necessarily represent those of their affiliated organizations, or those of the publisher, the editors and the reviewers. Any product that may be evaluated in this article, or claim that may be made by its manufacturer, is not guaranteed or endorsed by the publisher.
