# Supplementary material for: Nudix Hydroxylase 15 Mutations Strongly Predict Thiopurine-Induced Leukopenia Across Different Asian Ethnicities: Implications for Screening in a Diverse Population
Source: Front Med (Lausanne). 2022 Aug 5;9:880937. doi: 10.3389/fmed.2022.880937 (PMC9388767; doi:10.3389/fmed.2022.880937)
Supplement: Supplementary file 1 [file Data_Sheet_1.docx]

Manuscript Title: NUDT15 mutations strongly predict thiopurine-induced leukopenia across different Asian ethnicities

Authors: Xin-Hui Khoo, Shin Yee Wong, Nik Razima Wan Ibrahim, Ruey Terng Ng, Kee Seang Chew, Way Seah Lee, Zhi Qin Wong, Raja Affendi Raja Ali, Shahreedhan Shahrani, Alex Hwong Ruey Leow, **Ida Normiha Hilmi**

Supplementary details of SNPs Genotyping Assay

| SNP ID | Gene | Context Sequence [VIC/FAM] | Assay ID | Link To Product Information |
| --- | --- | --- | --- | --- |
| rs116855232 | NUDT15 c.415C>T | CCTGGACCAGCTTTTCTGGGGACTG**[C/T]**GTTGTTTAAAAGAACAAGGCTATGA | C_154823200_10 | <https://www.thermofisher.com/order/genome-database/details/genotyping/C_154823200_10?CID=&ICID=&subtype=#more-information-section> |
| rs186364861 | NUDT15 c.52G>A | GCGGCGGCCAGGAGTCGGAGTCGGA**[A/G]**TCGTGGTGACCAGCTGCAAGCATCC | C_181955856_10 | <https://www.thermofisher.com/order/genome-database/details/genotyping/C_181955856_10?CID=&ICID=&subtype=> |
| rs1142345 | TPMT c.719A>G | TCTCATTTACTTTTCTGTAAGTAGA**[C/T]**ATAACTTTTCAAAAAGACAGTCAAT | C_____19567_20 | https://www.thermofisher.com/order/genome-database/details/genotyping/C_____19567_20?CID=&ICID=&subtype= |
| rs1800460 | TPMT c.460 G>A | TCACCTGGATTGATGGCAACTAATG**[T/C]**TCCTCTATCCCAAATCATGTCAAAT | C__30634116_20 | https://www.thermofisher.com/order/genome-database/details/genotyping/C__30634116_20?CID=&ICID=&subtype= |
